# A blind spot? Confronting the stigma of hepatitis B virus (HBV) infection - A systematic review

**DOI:** 10.12688/wellcomeopenres.14273.2

**Published:** 2018-08-21

**Authors:** Jolynne Mokaya, Anna L McNaughton, Lela Burbridge, Tongai Maponga, Geraldine O'Hara, Monique Andersson, Janet Seeley, Philippa C Matthews

**Affiliations:** 1 Nuffield Department of Medicine, University of Oxford, Peter Medawar Building for Pathogen Research, South Parks Road, Oxford, OX1 3SY, UK; 2 Department of Gastroenterology, Oxford University Hospitals NHS Foundation Trust, Oxford, UK; 3 Department of Virology, University of Stellenbosch, Tygerberg Hospital, Bellville, Cape Town , 7500, South Africa; 4 Faculty of Infectious and Tropical Diseases, London School of Hygiene and Tropical Medicine, Keppel Street, London, WC1E 7HT, UK; 5 Department of Infectious Diseases and Microbiology, Oxford University Hospitals NHS Foundation Trust, John Radcliffe Hospital, Headley Way, Oxford, OX1 3SY, UK; 6 Department of Global Health and Development, London School of Hygiene and Tropical Medicine, Keppel Street, London, WC1E 7HT, UK; 7 Medical Research Council/Uganda Virus Research Institute and London School of Hygiene and Tropical Medicine, Uganda Research Unit, 51/59 Nakiwogo Rd, Entebbe, Uganda

**Keywords:** hepatitis B virus, discrimination, stigma, barriers, ethics, funding, elimination, Africa

## Abstract

**Background**: Stigma, poverty, and lack of knowledge present barriers to the diagnosis and treatment of chronic infection, especially in resource-limited settings. Chronic Hepatitis B virus (HBV) infection is frequently asymptomatic, but accounts for a substantial long-term burden of morbidity and mortality. In order to improve the success of diagnostic, treatment and preventive strategies, it is important to recognise, investigate and tackle stigma. We set out to assimilate evidence for the nature and impact of stigma associated with HBV infection, and to suggest ways to tackle this challenge.

**Methods**: We carried out a literature search in PubMed using the search terms ‘hepatitis B’, ‘stigma’ to identify relevant papers published between 2007 and 2017 (inclusive), with a particular focus on Africa.

**Results**: We identified a total of 32 articles, of which only two studies were conducted in Africa. Lack of knowledge of HBV was consistently identified, and in some settings there was no local word to describe HBV infection. There were misconceptions about HBV infection, transmission and treatment. Healthcare workers provided inaccurate information to individuals diagnosed with HBV, and poor understanding resulted in lack of preventive measures. Stigma negatively impacted on help-seeking, screening, disclosure, prevention of transmission, and adherence to treatment, and had potential negative impacts on mental health, wellbeing, employment and relationships.

**Conclusion**: Stigma is a potentially major barrier to the successful implementation of preventive, diagnostic and treatment strategies for HBV infection, and yet we highlight a ‘blind spot’, representing a lack of data and limited recognition of this challenge. There is a need for more research in this area, to identify and evaluate interventions that can be used effectively to tackle stigma, and to inform collaborative efforts between patients, clinical services, policy makers, traditional healers, religious leaders, charity organisations and support groups.

## Abbreviations

     •    PMTCT – Prevention of mother to child transmission

     •    TB – Tuberculosis

     •    HBV – Hepatitis B virus

     •    HCC – Hepatocellular carcinoma

     •    WHO – World Health Organization

     •    HIV – Human Immunodeficiency Virus

     •    HCWs – Healthcare workers

     •    THs – Traditional healers

     •    CHB – Chronic Hepatitis B

## Introduction

Stigma is recognised as a challenge in association with many infectious diseases, but has been poorly studied and is inadequately recognised for viral hepatitis infections. Hepatitis B virus (HBV) infection has been reported worldwide, with an estimated burden of 250–290 million cases, and has been associated with high mortality rates resulting from complications including cirrhosis and hepatocellular carcinoma (HCC) ^1, 2^. There are effective prevention and treatment strategies available, including vaccination and suppressive antiviral therapy, both of which also contribute to prevention of vertical transmission ^3^. The Global Hepatitis Health Sector Strategy is aiming for the elimination of viral hepatitis as a public health threat by 2030 ^4^. However, stigma, poverty, and lack of knowledge can be significant barriers to diagnosis, treatment and prevention, especially in resource-limited settings ^3^. These issues sit alongside additional challenges including gaps in vaccine coverage ^2, 5^, limited provision of diagnostic tests and treatment ^2^ and lack of a curative therapy ^3, 5^. There is a growing body of personal testimonies providing evidence of stigma associated with HBV ^6, 7^. However, there is a very limited literature to describe this. We have therefore used the metaphor of a ‘blind spot’ to describe the current situation for HBV stigma. This reflects a genuine gap in culture or society in which there is no word for the infection, it highlights the paucity of published data, and it describes a problem that is known to exist, but which is often neglected or ignored by current interventions, policy and practice. In light of this, more work is required to understand the nature and impact of stigma for individuals with HBV infection.

Individuals who are stigmatised as a result of illness or infection not only have to contend with potential challenges to health, but may also be denied the opportunities that define quality of life such as education, employment, access to appropriate health care and interaction with a diverse cross-section of society ^8^. Although many diseases are stigmatised, awareness of – and investigation into – stigma is better represented in some areas than others. For example, in HIV, stigma has been shown to limit engagement with services including screening and prevention of mother to child transmission (PMTCT), and uptake of antiretroviral therapy ^9– 12^; in tuberculosis (TB) it has been shown to cause diagnostic delays and treatment non-compliance ^13, 14^, and in mental illness, stigma has been associated with delays in help-seeking, discontinuation of treatment, suboptimal therapeutic relationships, patient safety concerns, and poorer quality mental and physical care ^15– 17^.

Stereotypes and prejudice may stem from physical differences attributable to the condition, misconceptions associated with fear of contagion, and judgements about routes of transmission, all of which may be underpinned by lack of knowledge ^18– 20^ and stigma is enhanced by poor of awareness, education and perception ^21^. The importance of physical signs of illness is exemplified for HIV, where those displaying wasting syndrome and certain identifiable opportunistic infections have been described as suffering more stigma compared to those who are asymptomatic ^9^. HIV and TB may also be stigmatised as a result of anxieties about spread of infection, and may be regarded as a punishment for ‘irresponsible’ or ‘immoral’ behaviour, ^14, 22– 24^. People with mental illnesses may be considered ‘dangerous’ or provoke fear, may lose their autonomy, or be treated as ‘childlike’ ^8^. Understanding why society reacts in a particular way is one way to address stigma ^19^, and could therefore help to guide approaches to tackling stigma in HBV.

HBV infection is highly endemic in Africa, but there are huge challenges in prevention, diagnosis and treatment ^2^. The situation is further complicated by the substantial public health challenge of co-endemic HIV and HBV ^25^. An understanding of the breadth and scope of stigma in HBV, especially in Africa, is an essential part of any strategy that seeks to tackle and eventually eliminate HBV infection as a public health problem. As the improvement of universal availability and accessibility of diagnostic, treatment and vaccination options are underway, it is important to ensure that products and services are not only available, but also accessible; people with HBV infection must be able to access education, clinical care, and support from their partners and families, healthcare workers, and members of their communities. The WHO recommends HBV treatment in an environment that minimizes stigma and discrimination ^26^.

We set out to assimilate evidence for the nature and impact of stigma on the lives of people with HBV infection and on the community, to describe coping strategies employed by people with HBV, and to suggest ways to tackle stigma and discrimination. Our approach was to gather relevant information regarding HBV stigma from the published literature using a systematic approach, to curate it in order to unify key messages, and to highlight gaps where future work is still needed. We have been able to develop suggestions to address HBV stigma and discrimination from the existing literature, but in light of the paucity of HBV-specific studies, we have also triangulated our approach by drawing on resources from other stigmatised conditions including HIV, TB and mental illness to inform the discussion.

## Methods

### Search strategy: systematic literature review

**Figure 1.**
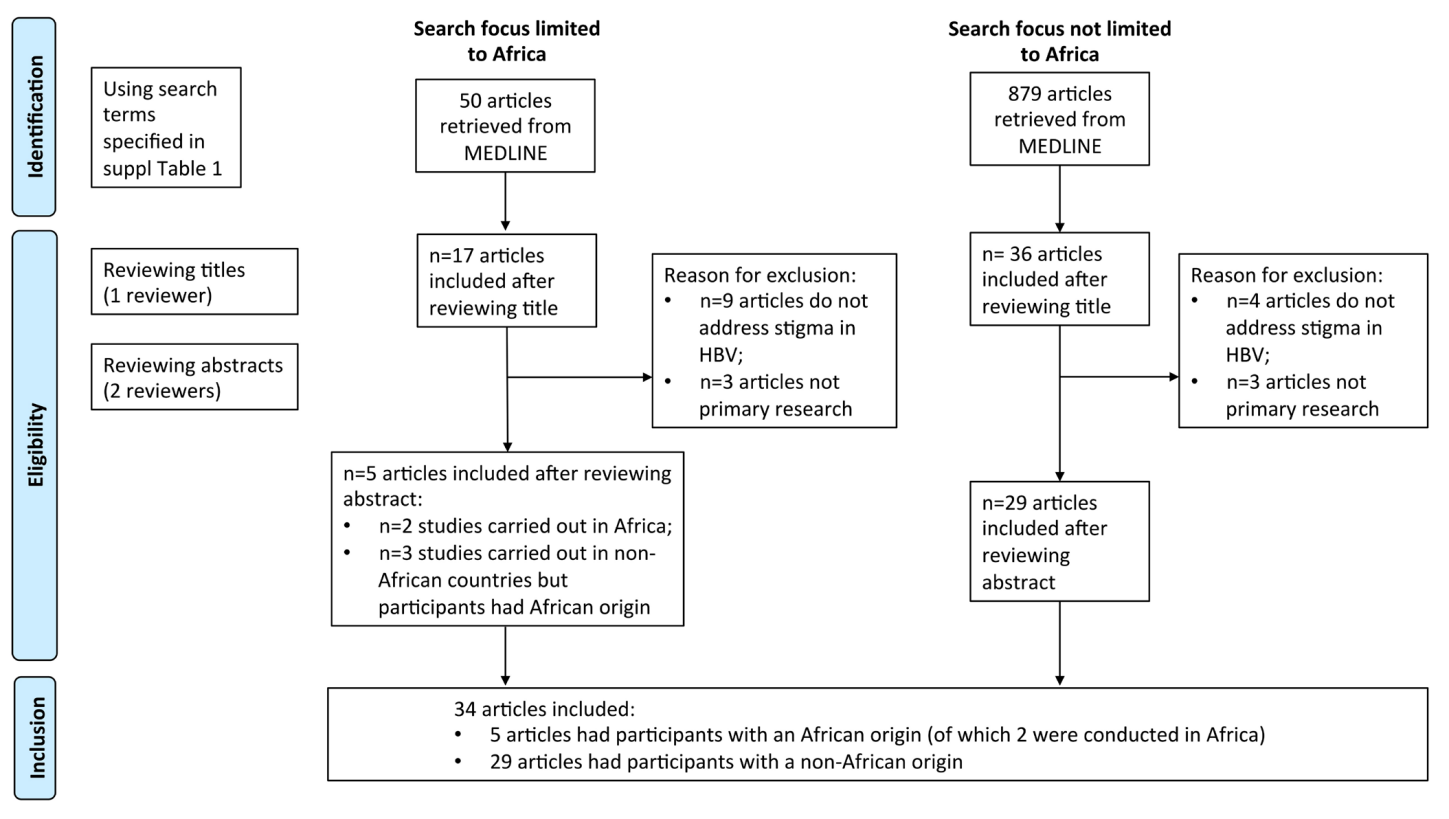
Flow diagram illustrating identification and inclusion of studies for a systematic review of stigma in HBV, based on PRISMA criteria

In November-December 2017, we undertook a systematic literature search in PubMed; our search strategy focused entirely on the evidence detailing stigma in HBV ( Figure 1). The terms of this search are detailed in Supplementary file 1. We carried out two searches: the first search focused on stigma in HBV in Africa, and a second search was not limited to Africa. The two searches yielded 50 and 879 articles, respectively (n=929). We removed 49 duplicate articles (n=880). On reviewing the titles and abstracts, 827 were excluded from search results as they were not on stigma or HBV. Full texts of 53 articles were reviewed by two different individuals, and 19 were excluded (6 articles not primary studies and 13 articles do not address stigma in HBV). A total of 34 articles were therefore downloaded in full. From each publication, we extracted the following: citation, study design, sample size, study population, country, factors associated with stigma, impact of stigma on the lives of people with HBV, and proposed interventions to tackle stigma in the society. We used our research question to group data collated from the included studies into four major themes: factors underpinning stigma in HBV infection, evidence for the impact of stigma, coping strategies for individuals with HBV infection, and interventions proposed to tackle stigma in HBV. There were insufficient data from Africa to focus specifically on that continent, but where possible we have highlighted issues that have been reported in African populations. Data were curated using MS Excel software (Microsoft, Redmond, WA). Ethics approval was not required for this study.

### Search strategy: other relevant resources

In order to inform a wider understanding of the potential relevance and impact of stigma in infection and illness, we have also referred to articles published on other conditions including HIV, TB and mental health to underpin hypotheses set out in the introduction, and to inform discussion. These papers were not identified through a formal systematic review, but were identified as relevant sources from robust, peer reviewed literature.

### Quality of evidence assessment

For the 32 studies included from our systematic review, we performed a risk of bias assessment ( Supplementary file 2). For qualitative studies (n=12) we used the qualitative appraisal checklist by NICE public health guidance ^27^ and for quantitative studies (n=19) we used the Centre of Evidence based Management checklist ^28^. One study used a mixed method study design ^29^, and we assessed this using each of the two approaches above. 

## Results

A full list of citations generated from our systematic literature review of HBV stigma is shown in Table 1. We have also provided an expanded version of this table, with details summarising the key information pertinent to stigma, in Supplementary file 2.

**Table 1.** Characteristics of 32 studies identified using a systematic literature search for stigma in HBV.

Citation (Author; Date; PMID), divided by region of origin and presented alphabetically by first author	Country where study took place	Study design	Study participants	Sample size	Recruitment site
AFRICA					
Adjei *et al.* 2017; PMID: 29102991 ^30^	Ghana	Qualitative	People with HBV infection	14	Hospital
Mkandawire *et al.* 2013; PMID: 23811012 ^31^	Ghana	Qualitative	Local chiefs, village elders and HCWs	72	Community
NORTH AMERICA					
Blanas *et al.* 2015; PMID: 25000917 ^32^	USA	Qualitative	West African Immigrants	39	Community
Carabez *et al.* 2014; PMID: 24395631 ^33^	USA	Survey	HBV positive Asian Americans	154	Community
Cheng *et al.* 2017; PMID: 27770375 ^34^	USA	Survey	Asian	404	Community
Cotler *et al.* 2012; PMID: 22239504 ^35^	USA	Survey	Chinese immigrants	N/A	Community
Frew *et al.* 2014; PMID: 25506280 ^36^	USA	Survey	Vietnamese Americans	316	Community
Li *et al.* 2007; PMID: 22993729 ^37^	Canada	Survey	Canadian Chinese	343	Community
Russ *et al.* 2012; PMID: 22440043 ^38^	USA	Qualitative	Asian Americans	HCWs:23 Individuals with HBV: 17	Hospital
Wu *et al.* 2009; PMID: 19172206 ^39^	Canada	Survey	People with HBV infection	204	Community
Yoo *et al.* 2012; PMID: 21748476 ^40^	USA	Qualitative	Asian American	23	Community
EUROPE					
Cochrane *et al.* 2016; PMID: 26896472 ^41^	UK	Qualitative	Somali community living in UK	30	Community
Lee *et al.* 2017; PMID: 28950835 ^42^	UK	Qualitative	Chinese immigrants	61	Community
Sweeney *et al.* 2015, PMID: 25890125 ^43^	UK	Qualitative	Immigrant communities and HCWs	118	Community and healthcare centres
van der Veen *et al.* 2014; PMID: 23913128 ^44^	Netherlands	Survey	Turkish-Dutch community	N/A	Community
MIDDLE EAST AND ASIA					
Dam *et al.* 2016; PMID: 28101498 ^45^	Vietnam and USA	Survey	General population	1012	Hospital
Eguchi *et al.* 2013; PMID: 24086765 ^46^	Japan	Survey	Japanese working community	3129	Community
Eguchi *et al.* 2014; PMID: 24792095 ^47^	Japan	Survey	General population	3000	Community
Huang *et al.* 2016; PMID: 27206379 ^48^	China	Survey	Individuals with HBV and healthy controls	1236	Community
Ishimaru *et al.* 2016; PMID: 27108645 ^49^	Japan	Survey	Nurses	992	Hospital
Ishimaru *et al.* 2017; PMID: 29165125 ^50^	Vietnam	Survey	Nurses	400	Hospital
Leng *et al.* 2016; PMID: 27043963 ^51^	China	Survey	General population	903	Community
Mohammed *et al.* 2012; PMID: 22856889 ^52^	Malaysia	Survey	People with HBV infection	483	Hospital
Ng *et al.* 2013; PMID: 21807630 ^53^	Malaysia	Qualitative study	People with HBV infection	44	Hospital
Rafique *et al.* 2015; PMID: 25664518 ^54^	Pakistan	Quantitive and qualitative study	People with HBV infection	140	Hospital
Taheri Ezbarami *et al.* 2017; PMID: 29085657 ^55^	Iran	Qualitative study	People with HBV infection	27	Community
Valizadeh *et al.* 2016; PMID: 26989666 ^56^	Iran	Qualitative study	People with HBV infection	18	Hospital
Valizadeh *et al.* 2017; PMID: 28362662 ^57^	Iran	Qualitative study	People with HBV infection	15	Hospital
Wada *et al.* 2016; PMID: 26850002 ^58^	Japan	Survey	Nurses	992	Hospital
Wai *et al.* 2005; PMID: 16124053 ^59^	Singapore	Survey	People with HBV infection	192	Community
Wallace *et al.* 2017; PMID: 28764768 ^60^	China	Qualitative study	People with HBV infection	41	Hospital
Yu *et al.* 2015; PMID: 26733133 ^61^	China	Survey	General population	6538	Community
AUSTRALIA					
Drazic *et al.* 2013; PMID: 23171324 ^62^	Australia	Survey	General population	77	Community
Sievert *et al.* 2017; PMID: 28120131 ^29^	Australia	Survey	Afghan, Rohingyan and Sudanese community	26	Community

**HBV = hepatitis B virus, HCW = healthcare worker**

### Quality of evidence

Asia and North America were best represented by the literature, in contrast to Africa from where we identified only two published studies, both set in Ghana ( Table 1). Among the 19 studies that used a quantitative study design, eight used a convenient sampling method which introduces selection bias ^33– 35, 37, 45, 51, 59, 62^, two studies did not describe a sampling method ^39, 58^, five used a random sampling method ^36, 46, 47, 50, 61^, three studies included all intended participants ^44, 49, 52^, and only one study used a sample size based on pre-study consideration ^47^. A participant response rate was presented in 10 studies; in nine of these the response rate was >60% ^35– 37, 45, 49– 52, 59^ and one had a 30% response rate ^44^. Statistical significance of results was reported in 15/19 studies ^34– 37, 39, 44– 48, 51, 52, 59, 62^. 

All the qualitative studies described how concepts and themes were derived, presented findings clearly using extracts from original data and their findings were relevant to the research question ^30– 32, 40– 43, 53– 56, 60^. All except two of these ^40, 41^ clearly described data collection procedure and the research context. Analysis of data may be less robust in four studies ^41, 42, 55, 56^ since there was no description of how many researchers analysed the results and how differences in extracted themes and codes were resolved between researchers. Two studies did not provide details of ethics approval ^40, 42^; three studies did not provide a rationale for using the selected study methodology ^40, 43, 60^; seven studies did not report in detail on their study limitations ^29, 42, 43, 53, 55, 56, 60^. For the single study that used mixed methodology ^29^, a convenient sample was used. Participants’ response rate and statistical significance of quantitative data was not reported. In the qualitative arm, data collection and analysis was clearly described but it was not stated how many researchers analysed the data and how discrepancies were handled.

There was considerable diversity in the size of the population sampled (range 14–6538), but overall, most studies had small sample sizes (median 173), and some were opportunistic in recruiting participants, thus limiting the generalisability of the findings.

### Themes

The data that we assimilated from our literature review of 32 systematically identified studies are presented in Table 2– Table 4 below.

**Table 2.** Factors underpinning stigma in HBV infection, identified from a systematic literature review.

Factor underpinning stigma	Evidence from systematic literature review
**Cultural understanding and relevant** **language**	• Lack of common cultural understanding of HBV is indicated by an absence of any word in local languages to define HBV ^31, 41^ and missing terms ‘hepatitis B’ and ‘carcinoma’ from English vocabulary in migrant populations ^29, 41^; • There is confusion between HBV and other infections, including malaria, yellow fever and HIV ^31, 32^; HBV may be seen as synonymous with ‘jaundice’ ^29, 41, 43^, or believed to be associated with nutritional status ^41^; • One study describes the assumption that hepatitis A, B and C infections are ranked by letter in order of severity, or represent the chronological development of a single infection ^43^.
**Knowledge about diagnosis,** **treatment and symptoms of chronic** **HBV infection**	• Poor understanding of the chronic nature of HBV infection, and lack of insight into the asymptomatic nature of HBV infection and its complications, are well described ^32, 39, 42, 43, 53, 59^; • There may be an assumption that lack of symptoms correlates with lack of severity ^29^; • Poor awareness of treatment options can be associated with a ‘passive’ or ‘fatalistic’ attitude towards treatment ^32, 53^; • There are misconceptions that HBV screening tests can be harmful and that HBV infection is not treatable ^36^; • A significant correlation is reported between less knowledge and higher stigma scores ^34^. However, among individuals with HBV infection, higher levels of HBV knowledge can also be associated with being more worried ^32, 41, 52^; • Improved knowledge of HBV infection is associated with higher levels of formal education ^39, 52, 59, 61^, and with a close relationship with an individual infected with HBV ^35^.
**Beliefs and insights into** **transmission of HBV infection**	• Beliefs are widespread that HBV can be transmitted through sharing of utensils, via food and water, or eating together ^33– 35, 37, 39, 41, 43, 45, 48, 51– 53, 59^; • There is a belief that smoking tobacco causes HBV ^36^; • Some studies report beliefs that HBV infection arises as a result of poor sanitation ^41, 43^ or could be transmitted by sharing water for bathing ^46^; • In some communities, HBV infection is regarded as a genetic trait ^34, 56^; • HBV infection is represented as a consequence of immoral behaviour ^40, 42– 44^, or as a punishment for sins ^55^; • Some communities believe that HBV infection is caused by witchcraft or evil spirits; this can be associated with pursuit of traditional remedies or religious interventions ^29– 31^; • Poor insights into transmission are associated with lack of precautions for prevention of transmission ^35, 56^; • Awareness of injecting drug use and sexual transmission of HBV can be stigmatising ^32, 42^.
**Sociodemographic factors**	• In some studies older age has been associated with increased stigma ^45, 48, 61^; however, this is not consistent, as older age has also been associated with decreased levels of stigma ^47^; • People strongly defined by traditional values are more likely to stigmtise HBV infection ^55^; • Unemployed individuals from rural areas are more likely to experience discrimination ^51, 60^; • HBV may be more prevalent in disadvantaged groups who are also stigmatised for other reasons, eg refugees ^29^; • Stigma can be reduced by having a family member with HBV infection; in this case it clarifies misconceptions about the disease, humanizes the affected, and can reduce negative attitudes associated with cultural beliefs ^34, 35^.
**Interactions with HCWs**	• Lacking or inaccurate information may be provided by HCWs regarding HBV diagnosis or treatment, including inappropriate reassurance, or overemphasis of potential complications ^49, 53^; • Some studies describe HCWs expressing discrimination or prejudice towards patients and/ or colleagues with HBV infection ^46, 58^, which may be more common among those who have poor knowledge or are unfamiliar with providing HBV care ^49^; • Diagnosis of HBV infection is presented as ‘bad news’ which can add to anxiety and stigma ^53, 56^; • Lack of screening or vaccination may be associated with stigma ^34^, although in contrast, HBV-related discrimination is also described as arising in association with diagnostic screening ^51^.
**Emotional responses**	• HBV infection can be associated with anxiety, fear and depression; see Table 3 for further details and references.

**Table 3.** Evidence for the behavioural, psychological and social impact of HBV infection, identified from a systematic literature review.

Factor with impact on HBV infection	Evidence from systematic literature review
**Access to** **appropriate** **health care** **and treatment** **compliance**	• Stigma is associated with reduced uptake of opportunities for diagnostic screening and clinical care ^35, 37, 38, 42, 48, 56, 57^; • There is a low rate of disclosure of HBV status among individuals with HBV to family, other acquaintances, and to HCWs due to fear of stigma ^48, 55^; • Stigma can lead to disengagement from treatment ^49^ and reduce treatment adherence ^55^; • Anxiety about treatment, or the cost of treatment (potentially not just for one individual but also for other family members), can lead to reluctance to seek clinical care and follow-up ^32, 49, 60^; • Treatment can be seen as futile ^55^; • Stigma can lead to negative experiences of health care such as being ‘labelled’, placed in physical isolation, or criticised by HCWs ^55^; • Stigma may influence the priorities of health professionals and commissioners, leading to certain health issues being addressed whilst others are ignored ^42^.
**Impact on** **opportunities** **for education,** **work and career** **development**	• Discrimination is reported within schools ^35, 45^; • HBV affects employment and education choices ^48, 60^; individuals with HBV infection may be discriminated against at work, lose employment, or be unable to find work ^29, 34, 42, 54– 56, 60^; they may also be concerned about missing work due to illness ^33^, and may be restricted from particular jobs (e.g. food preparation) ^37, 52^; • As a result of discrimination in education and the work-place, HBV can prevent personal goals or potential from being fulfilled ^55^.
**Impact on mental** **health**	• People with HBV infection may fear physical consequences of transmission, disease progression and/or treatment side effects ^29, 51, 53^, including fear of cancer and death ^30, 33, 55, 56^; • Individuals in migrant populations may fear deportation ^32, 38^; • A range of emotional responses is described, included shock and grief following initial diagnosis, and subsequently anxiety, sadness, denial, anger and aggression ^30, 52, 56, 57, 62^; • Negative self-image can be associated with infection, associated with feelings of disgrace, guilt and shame, humiliation, embarrassment, or inferiority ^32, 34, 35, 39, 44, 48, 52, 53, 56^; • Reactions can also include insomnia and depression ^49, 53– 56, 60^, and suicidal ideation ^42, 54^; • Anxiety is described in association with the economic cost of treatment ^29^; • A fear of disclosure promotes secrecy and isolation ^55, 56, 60, 62^.
**Impact on** **personal** **interactions**	• ‘Fear of contagion’ causes isolation; individuals with infection either avoid or are rejected from social activities, including avoidance of sharing utensils / food / towels / soap ^35, 37, 42, 43, 48, 51, 53, 54, 56, 57, 60^, and may be banned from participation in sports ^55^; • Parents may be unwilling to allow their children to socialise with HBV-infected children ^51, 61^; • Anxiety about spreading infection to family members leads to withdrawal from close relationships ^29, 32, 35, 52, 53, 57^; and exclusion from social gatherings ^60^; • Individuals with HBV infection perceive themselves, or are perceived by others, as less desirable as a parent or spouse ^33– 35, 45, 48, 54^; • Partner or spouse refuses sexual intercourse or insists on use of condoms ^54^; • Individuals with HBV infection may seek assistance from traditional healers (THs) or faith leaders, seeking ‘purification’; although this can provide important psychosocial support, there is also the potential for harm and/or delaying presentation to clinical care ^30^.

**Table 4.** Interventions that have been used to tackle stigma in HBV, identified from a systematic literature review.

Category of intervention	Evidence from systematic literature review
**Interventions targeting** **individuals with HBV** **infection**	• Provision of educational opportunities in health care settings, in the individual’s own language, can be valuable to inform patients of the importance of symptoms, treatment, follow-up, prevention and social stigma ^32, 33, 43, 52^; • Improving knowledge regarding the potential for silent complications, and understanding treatment could enhance willingness to access healthcare and reduce fatalism ^32, 39^; • Developing positive coping strategies may include seeking encouragement from spiritual leaders and open dialogue with family members ^33, 43, 48, 55^; • Lifestyle modifications can be helpful, such as reduced intake of alcohol and fatty foods ^30, 33, 52^; • Barriers to interventions should be considered (e.g. remote location, no internet access, language barriers) ^62^.
**Interventions targeting** **HCWs**	• Education and training for HCWs should be improved, particularly for community doctors in rural settings ^61^; this includes training in diagnosis, prevention, treatment and monitoring ^31, 33, 35, 37, 49, 53^, and provision of appropriate pre- and post-test counselling ^29, 33, 49^; • HCWs can be deployed to design interventions to encourage participation in treatment programs and better self-care ^55^; • Education and communication programmes are needed to reduce stigma towards patients and colleagues with HBV infection ^49– 51^; • Championing a positive safety culture, such as strict infection control, may not only protect HCWs but can also improve the quality of patient care ^50^.
**Interventions targeting** **communities and policy** **makers**	• Culturally appropriate education programs are required to improve knowledge on symptoms, modes of transmission and preventative measures, to correct misconceptions and decrease stigma; this could include use of internet, social media, radio, posters in public places (bars, markets, healthcare settings), and should involve schools and religious leaders ^31– 36, 40, 41, 47– 49, 53, 55^; • Specialised training on HBV for THs and ministers of faith could be a valuable approach ^30^; • Targeting individuals directly (e.g. with letters) might be of greater benefit than mass media campaigns ^41^; • Developing national campaigns to promote HBV screening and vaccination as positive strategies that can empower local communities in their own healthcare ^40, 53^; • Establishing a human connection can increase familiarity and reduce apprehension and negativity ^42^; for example, high profile public advocates have been successful in the ‘B Free Campaign’ ^34, 40^; • National strategic responses to HBV infection should specifically acknowledge and address the social implications of the infection ^60^, and should prioritise affordability of prevention and treatment ^36^.

## Discussion

Through this review, we have been able to identify some key themes including the potentially profound impact of HBV infection on personal wellbeing and mental health, opportunities for education and career, influence on family and relationships, and the role that health care workers can play in either contributing to stigma or dispelling it. Through enhancing consistent insights into these areas, the ‘blind spot’ associated with HBV stigma can start to be reduced. Previous reviews addressing stigma in Asian populations have reported small numbers of relevant publications, but identify the challenges of stigma, poor knowledge, and lack of focus on appropriate interventions ^63, 64^. This review demonstrates the lack of data for the whole of the African continent which is particularly striking. A recent report showed that in Africa, hepatocellular carcinoma (HCC) secondary to HBV infection frequently presents at a late stage, where effective treatment would be challenging for any healthcare system ^34^, and indeed presentation is too late to allow formal diagnosis or treatment – this illustrates a hidden burden of infection, another aspect of the ‘blind spot’.

### Stigma associated with physical disease manifestations

In most cases, HBV infection is asymptomatic and invisible for many decades, so individuals are not likely to be physically stigmatised. However, in acute infection, and again in the latter stages of chronic disease, it may cause jaundice and other manifestations such as abdominal swelling from ascites. We have not found any representation in the literature of stigma associated with specific physical syndromes, but further work is needed to explore this possibility. Importantly, health-related quality of life, including mental health, has been shown to decline in the setting of more severe manifestations of HBV infection ^65^.

### Relationship between stigma and knowledge or education

Findings in Africa demonstrate a general lack of knowledge on HBV, an inability to define the disease, confusion of HBV with other infections, including malaria, yellow fever and HIV ^31, 32^, and the association of HBV symptoms with witchcraft or poisoning ^30, 31^. Such negative associations have been described to have the potential to ‘spoil the identity’ of the individual: the individual ceases to be perceived as a normal person but rather one who is tainted and discounted from society ^20^. Lack of knowledge or awareness leads to misinformation that feeds stigma and discrimination ^66^. Formal education can be important in reducing discrimination and stigma ^46^; however, it is interesting to note that this is not always the case. Indeed, higher levels of knowledge or formal education can sometimes be associated with increased stigma ^39, 46^. One study describes how specific knowledge - such as recognising sexual contact as a transmission route – can actually increase stigma ^32^. In other settings, education level did not have a specific relationship with stigma ^51^. A general lack of stigma was reported associated with HBV in the Somali population, but this study reports that the problem of stigma is increasing over time as education increases ^41^.

Age may be relevant to knowledge or education, and varying influence of age on stigma has also been reported; in some instances older age and traditional values have been associated with stigma ^45, 46, 53^ while in others, there is decreased prejudice among older age ^47^. In light of these discrepancies, it is important to design studies with bigger sample sizes and conduct studies in different regions and communities. This will help to not only provide a clear explanation of factors associated with stigma in HBV but will also guide in designing interventions that target specific groups of people who might be most vulnerable to the effects of stigma.

### Interventions for HBV stigma

Depending on the coping strategies used, not everyone experiencing stigma will necessarily suffer emotional distress or have diminished well-being as a consequence ^67^. In HIV, avoidant coping strategies such as denial have been associated with higher levels of depression whereas acceptance of the diagnosis, associated with personal control and self-efficacy, is associated with reduced levels of depression ^68^. Among patients with schizophrenia, the ability to use positive coping strategies was associated with reduced self-stigma ^52^. Stigma and/or the disease process can contribute to the decline of health-related quality of life and mental health of some individuals living with chronic HBV ^65^, however there are few studies that address these issues. In light of this, further studies are needed to explore coping strategies used to deal with HBV stigma, to explore the choice of coping strategy, and to determine outcomes.

As well as individual coping strategies, a variety of other interventions have been proposed to tackle stigma ( Table 3). In many cases, attitudes may improve after realising that infection can be prevented and managed therapeutically ^37^. This is in line with a report from the WHO about mental illness, highlighting that stigma can be combated through educational messages representing conditions as illnesses that respond to specific treatment ^46^. Counselling can provide several benefits, alleviating anxiety associated with a new diagnosis, as well as providing an opportunity to share factual information regarding treatment, prevention, self-care and overall well-being ^33, 49^. This should be delivered sensitively, at a time and in a manner that supports the individual in accepting and processing the information.

There are several strategies that have been used to raise awareness of HIV, many of which could be applied to HBV. Dissemination of factual information through use of radio, television, posters, pamphlets and drama has been widely used to diminish stigma towards HIV in resource limited settings ^9^. Multiple educational sessions can also be an effective way of increasing awareness and therefore reducing stigma, as participants have the opportunity to reflect on the concepts learnt in the previous sessions ^58^. Another successful method for training HCWs is perspective-taking and empathy: participation is associated with ‘increased willingness to treat people with certain illnesses, decreased stigma and increased awareness of confidentiality among healthcare workers’ ^58^. These approaches could be used to tackle HBV stigma among HCWs and community members.

On-line support groups provide a platform for people living with HBV to share their experiences ^69, 70^, and charities can be influential in raising awareness, and promoting important health messages ^70^. The internet is widely used as a tool for sharing experiences, and as such can raise awareness of stigma ^7^, as well as uniting individuals and communities as a support network. It is important for HCWs to introduce newly diagnosed individuals to these avenues to help people living with HBV. Importantly, more charities and support groups that are well suited for people with HBV infection in Africa are needed.

### Limitations and caveats

Only two studies were identified on HBV stigma from Africa ^30, 31^; these studies were both carried out in one country (Ghana). Three other studies included African participants in USA ^32^, Australia ^29^ and in UK ^41^. This highlights the substantial problem of HBV neglect in Africa. However, although we undertook a robust systematic search of the peer-reviewed scientific literature, there may be other sources that are not captured by this scientific style of approach.

Sub-Saharan Africa encompasses huge diversity, and as such it would be misleading to assume that we can generalise about culture, beliefs and stigma, or that findings that arise in one setting can be extrapolated to others; the knowledge, manifestations, and experience of stigma is bound to be different between settings. It is frequently the case that local, indigenous knowledge and understanding of disease and/or health conditions is not well reflected in the published literature. Further work will be needed to develop insights that are relevant to particular populations in order to develop the most effective interventions. Referring to specific examples for particular places or countries is currently difficult in the absence of more published data. However, to understand some of the specific challenges of HBV, we have previously collated individual experiences of HBV from patients, researchers and healthcare workers representing different settings across sub-Saharan Africa ^3^.

Our description of ‘coping strategies’ is an over-simplification of the complex responses that arise as a result of stigma. We recognise that many individuals who suffer stigma may not be able to deploy specific active coping mechanisms, and indeed may ‘endure’ their situation.

### Future aspiration and challenges

We see this article as a starting point for this field, as it is currently very difficult to make substantial advances in the absence of better data. By collating the existing literature, we hope to raise the profile of this topic overall, to improve recognition of the problem, to promote dialogue, and to highlight specific areas of neglect. This provides a foundation for healthcare workers and researchers to build on over time.

Studies looking at stigma may benefit from a mixed method study design, providing stronger evidence and increasing generalisability of the findings. It is important to carry out research on stigma with the aim of demonstrating its burden and its effects, while also considering how to establish and evaluate interventions that tackle stigma within communities. We identified only one study that evaluated the effectiveness of stigma reduction programmes among Asians in USA ^40^. More studies like this are needed in Africa, in order to provide insights into local understanding and beliefs, as they will also help to deploy resources in the most appropriate and effective ways with particular relevance to individual settings. In addition to raising awareness among individuals with HBV infection, the general public and HCWs, enhanced communication with policy makers is also crucial. Despite the high burden of HBV in low and middle-income countries, there is limited infrastructure to support diagnosis and treatment, and disproportionately little international research funding for HBV compared to other infectious diseases ^3^.

## Conclusion

Despite the limited evidence from Africa, the data we have gathered reflect consistent themes in stigma and discrimination affecting individuals with chronic viral hepatitis infection. These may have a wide-reaching influence on physical health (for example inhibiting interaction with clinical services and reducing treatment adherence), psychological well-being (through increasing isolation, anxiety and depression), and interactions with family and the wider society (by limiting relationships, social interactions and employment opportunities). Recognising, understanding and tackling the issue of this stigma in African populations could be a valuable tool to improve population health and to underpin advances towards elimination strategies proposed for the year 2030. Education clearly provides a very important role in reducing discrimination and stigma; it is interesting to note that stigma may drive lack of knowledge, just as lack of knowledge may drive stigma.

There is also a pressing need for more research in this area, to identify and evaluate interventions that can be used effectively to tackle stigma in HBV, and for collaborative efforts between policy makers, HCWs, traditional healers, religious leaders, charity organisations and support groups, to improve awareness and tackle stigma in HBV in Africa.

## Data availability

All data underlying the results are available as part of the article and Supplementary files, and no additional source data are required.

## Supplementary material

**Supplementary file 1: Details of search strategy used to identify studies on stigma in Hepatitis B virus (HBV) infection, from PubMed database.** The terms in each row were combined by Boolean operator ‘OR’, the columns were combined by Boolean term ‘AND’. We carried out two searches: the first search focused on stigma in HBV in Africa - we combined all the columns in search strategy (#1 AND #2 AND #3); a second search was not limited to Africa – for this we only combined columns (#1 AND #3).

**Supplementary file 2: Full details of 32 studies identified by a systematic literature search of stigma in Hepatitis B virus (HBV) infection published between 2005 and 2017.**

**Supplementary file 3. Details of risk of bias assessment, using Centre of Evidence based management checklist, on 14 quantitative studies identified by a systematic literature search of stigma in Hepatitis B virus (HBV) infection published between 2005 and 2017.**

**Supplementary file 4. Details of risk of bias assessment, using NICE public health guidance qualitative appraisal checklist, on 20 quantitative studies identified by a systematic literature search of stigma in Hepatitis B virus (HBV) infection published between 2005 and 2017.**
