# Supplementary material for: A blind spot? Confronting the stigma of hepatitis B virus (HBV) infection - A systematic review
Source: Wellcome Open Res. 2018 Aug 21;3:29. Originally published 2018 Mar 16. [Version 2] doi: 10.12688/wellcomeopenres.14273.2 (PMC6234740; doi:10.12688/wellcomeopenres.14273.2)
Supplement: Supplementary file 1 [file wellcomeopenres-3-16079-s0000.tgz › d732536a-b88e-4c2c-b91e-549e9bf65fc7.docx]

| **Problem (#1)** | **Region (#2)** | **Area of focus (#3)** |
| --- | --- | --- |
| Stigma OR stigmati* OR Stereotyping OR Social Isolation OR discriminat* OR prejudice* OR “social* exclus*” OR “Social Stigma” OR “Social Marginali*” OR “Social Discrimination” OR ignoran* OR misconception* OR misperception* OR shame | Africa[MeSH] *OR* africa[tiab] *OR* "sub Saharan africa"[tiab] *OR* "sub-Saharan Africa"[tiab] *OR* Angola[tiab] *OR* Benin[tiab] *OR* Botswana[tiab] *OR* "Burkina Faso"[tiab] *OR* Burundi[tiab] *OR* Cameroon[tiab] *OR* "Cape Verde"[tiab] *OR* "Central African Republic"[tiab] *OR* Chad[tiab] *OR* Comoros[tiab] *OR* "Republic of the Congo"[tiab] *OR* "Democratic Republic of the Congo"[tiab] *OR* "Cote d'Ivoire"[tiab] *OR* Djibouti[tiab] *OR* "Equatorial Guinea"[tiab] *OR* Eritrea[tiab] *OR* Ethiopia[tiab] *OR* Gabon[tiab] *OR* "The Gambia"[tiab] *OR* Ghana[tiab] *OR* Guinea[tiab] *OR* "Guinea-Bissau"[tiab] *OR* Kenya[tiab] *OR* Lesotho[tiab] *OR* Liberia[tiab] *OR* Madagascar[tiab] *OR* Malawi[tiab] *OR* Mali[tiab] *OR* Mauritania[tiab] *OR* Mauritius[tiab] *OR* Mozambique[tiab] *OR* Namibia[tiab] *OR* Niger[tiab] *OR* Nigeria[tiab] *OR* Rwanda[tiab] *OR* "Sao Tome and Principe"[tiab] *OR* Senegal[tiab] *OR* Seychelles[tiab] *OR* "Sierra Leone"[tiab] *OR* "South Africa"[tiab] *OR* "South Sudan"[tiab] *OR* Sudan[tiab] *OR* Swaziland[tiab] *OR* Tanzania[tiab] *OR* Togo[tiab] *OR* Uganda[tiab] *OR* Zambia[tiab] *OR* Zimbabwe[tiab] | Hepatitis B virus[Mesh] *OR* "hepatitis b" *OR*  HBV |

**Suppl data Table 1: Details of search strategy used to identify studies on stigma in Hepatitis B virus (HBV) infection, from PubMed database.**
