# Supplementary material for: A blind spot? Confronting the stigma of hepatitis B virus (HBV) infection - A systematic review
Source: Wellcome Open Res. 2018 Aug 21;3:29. Originally published 2018 Mar 16. [Version 2] doi: 10.12688/wellcomeopenres.14273.2 (PMC6234740; doi:10.12688/wellcomeopenres.14273.2)
Supplement: Peer review history

# Peer review: Referee response for version 2

**DOI:** 10.21956/wellcomeopenres.16079.r33738

**Published:** 2018-11-09

**Authors:** Herbert Muyinda

**Affiliations:** 1 Child Health and Development Centre, College of Health Sciences, Makerere University, Kampala, Uganda

I have read the revised copy of “A blind spot? Confronting the stigma of hepatitis B virus (HBV) infection - A systematic review”. Considerable revisions have been made, and I am convinced that it makes a contribution towards a debate on the Hepatitis B – related stigma, and further research on the topic. I believe and confirm that it is of an acceptable scientific standard, and should be indexed.

I have read this submission. I believe that I have an appropriate level of expertise to confirm that it is of an acceptable scientific standard.

# Peer review: Referee response for version 2

**DOI:** 10.21956/wellcomeopenres.16079.r33737

**Published:** 2018-10-04

**Authors:** Laura Nyblade

**Affiliations:** 1 RTI International, Washington, DC, USA

I have read this submission. I believe that I have an appropriate level of expertise to confirm that it is of an acceptable scientific standard.

# Peer review: Referee response for version 1

**DOI:** 10.21956/wellcomeopenres.15534.r32085

**Published:** 2018-06-05

**Authors:** Herbert Muyinda

**Affiliations:** 1 Child Health and Development Centre, College of Health Sciences, Makerere University, Kampala, Uganda

1. Are the rationale for, and objectives of, the Systematic Review clearly stated?

***Yes:*** The article addresses the important health aspect of HBV stigma. It states that there is minimal recognition of the challenge stigma poses especially in Africa, yet it is a potentially major barrier to the successful delivery and access to care. The authors set out to fully understand [assimilate] the evidence for the nature and impact of stigma on the lives of people with HBV infection, and on the community, and to suggest ways to tackle stigma and discrimination.

  

1. Are sufficient details of the methods and analysis provided to allow replication by others?

***Partly: ***This was a systematic literature review using a search in PubMed focusing on information published between 2007 and 2017, with a particular focus on Africa. However, although the target area of analysis was ‘Africa’, in the limitations section they state that there were only two studies conducted in Africa, in fact in the abstract they state that it was only one. This paucity of published information on the subject matter would call for triangulation – additional sources of information would be sought to supplement the one or two published articles reviewed.    

  

1. Are the conclusions drawn adequately supported by the results presented in the review?

***Partly: *** While this topic is important and merits careful analysis, this article presents over generalized already known assertions regarding stigma, with hardly new contribution to the topic. The authors approach the topic by posing a question in their title: “A blind spot? …” The implication here is that the article should explicitly articulate the nature of ‘blindspotness’ of HBV stigma. Non-availability or presence of few articles of published work on this particular topic may not be enough to make it a blind spot. To satisfy such a claim, it would be useful to address some of these questions: Do people (particularly in Africa) know what Hepatitis B disease is? What are some of the local names/labels/terminologies used in describing it? What are the individual and collective experiences of the disease, and what are the common social responses? In other words, a more detailed description and analysis of how stigma manifests itself and experienced by the affected individuals (in Africa) would be the way to illustrate the social (in)visibility and/or extent/magnitude of the ‘blindspotness’ of HBV stigma, as referred to in the article.

 First of all, focusing on Africa as a whole (as a target population) is one of the main challenges of the paper. Africa is too diverse to have such generalized ‘lack of knowledge’, ‘misconceptions and prejudices’ regarding HBV. The Moslem North and part of west Africa, the Sub-Saharan Africa, and the relatively wealthy South Africa are different cultures and the knowledge, manifestations, and experience of stigma is bound to be different. The paper can benefit from referring to examples, references in particular places or countries.

 While the article states the intention to assimilate the available evidence for the nature and impact of stigma on the lives of people with HBV infection and on the community, this does not seem to have been effectively achieved. The paper states very generalized and quite obvious views, which makes it difficult to find new contribution the paper makes to the topic of HBV stigma.

 There is a general tendency to be unspecific regarding manifestation and experience of stigma, by referring to experiences in other conditions – HIV and AIDS, and mental illness, and areas outside Africa. For instance, the authors emphasized lack of knowledge of HBV among different players (health workers, people with HBV infection themselves, community) as potent driver of stigma, and then link the negative effects thereof to prevention, treatment seeking and provision of care. On pg. 3 the authors state that “stereotypes and prejudice may stem from physical differences attributable to the condition, misconceptions associated with fear of contagion, and judgements about routes of transmission, all of which may be underpinned by lack of knowledge … arises from lack of awareness, lack of education and lack of perception … and that healthcare workers provided inaccurate information to those diagnosed by HBV.” While these assertions may be true, the kind of ‘knowledge’ being referred to as lacking in this case, is not clear. First, if there was ‘lack of knowledge’, then what would shape the HBV stigma stated in the paper? The fact that there was stigma, means that there was some form of knowledge; probably ‘inappropriate’ but some knowledge. Quite often, there is a tendency to disregard local/indigenous (African) knowledge and understanding of disease and/or health conditions including HBV, and emphasis placed on the biomedical knowledge and quoting sources from the Western World.

 Further, generally stigma is known to be characterized by labelling, and social constructs and actions that discount one’s identity (see Goffman: Notes on spoilt Identity). For example, it is not clear what knowledge of HBV bodily signs and symptoms people have, their local names/labels/terminologies, and how this knowledge shapes different forms of stigma. All these seem to have been missing in the literature, which makes it difficult to conclude from the review that people with HBV (in Africa) were actually being stigmatized, leave alone HBV stigma being a blind spot. In other words, the paper does not explicitly show whether or how HBV related stigma (in Africa), is blind spot.

**Other comments: **

- Coping strategies and interventions for HBV stigma” (Table 3)
  The coping strategies listed in the table and discussed in the paper assume existence of particular forms of stigma, that have not been shown to be evident or experienced in Africa. The paper can be improved by linking particular ‘coping’ strategies to particular forms of stigma (in Africa). In fact, in some cases the stigmatized hardly cope, but just endure, and the boundary between coping, and endurance is so blurred. 

Discussion:  

 The claim in the paper, that the data gathered reflect consistent themes of stigma and discrimination … is not adequately illustrated in the results section. Most of the examples given were from Asia and other areas other than Africa, and of other conditions – mental illness and HIV and AIDS, and not HBV, which hardly refelects any consistency at stated.  

Limitations:

 The limitations as stated, considerably weakens the paper. Given the paucity of data, a ‘blind spot on HBV stigma in Africa would be verified by triangulation of information clearly showing what is (in)visible/blindspot, rather than simply interpreting it as lack of literature on the subject matter.

I have read this submission. I believe that I have an appropriate level of expertise to confirm that it is of an acceptable scientific standard, however I have significant reservations, as outlined above.

# Response

**Published:** 2018-08-14

**Authors:** Philippa Matthews

**Affiliations:** Nuffield Department of Medicine, Peter Medawar Building for Pathogen Research, University of Oxford, UK

REVIEWER: Are sufficient details of the methods and analysis provided to allow replication by others? ***Partly: ***This was a systematic literature review using a search in PubMed focusing on information published between 2007 and 2017, with a particular focus on Africa. However, although the target area of analysis was ‘Africa’, in the limitations section they state that there were only two studies conducted in Africa, in fact in the abstract they state that it was only one.

 RESPONSE: We have corrected this statement in the abstract, this now reads” We identified a total of 32 articles, of which only two studies were conducted in Africa”. As per responses to reviewer 1, this information has also been presented more clearly in the form of a new table 1.

 REVIEWER: This paucity of published information on the subject matter would call for triangulation – additional sources of information would be sought to supplement the one or two published articles reviewed.    

 RESPONSE: We agree that additional sources of information are required, and this provides the justification for drawing on the literature for HIV, TB and mental health to provide more insights than can be gained from the HBV literature alone. In response to comments from reviewer 1, we have adopted a clearer stance on which sections of the manuscript draw on just the HBV literature, and which sections have included reference to wider sources. Please see our comments to reviewer 1 for more details.

 REVIEWER: While this topic is important and merits careful analysis, this article presents over generalized already known assertions regarding stigma, with hardly new contribution to the topic. The authors approach the topic by posing a question in their title: “A blind spot? …” The implication here is that the article should explicitly articulate the nature of ‘blindspotness’ of HBV stigma. Non-availability or presence of few articles of published work on this particular topic may not be enough to make it a blind spot. To satisfy such a claim, it would be useful to address some of these questions: Do people (particularly in Africa) know what Hepatitis B disease is? What are some of the local names/labels/terminologies used in describing it? What are the individual and collective experiences of the disease, and what are the common social responses? In other words, a more detailed description and analysis of how stigma manifests itself and experienced by the affected individuals (in Africa) would be the way to illustrate the social (in)visibility and/or extent/magnitude of the ‘blindspotness’ of HBV stigma, as referred to in the article.

 RESPONSE: We believe our work is a new contribution to the topic, as no prior attempt has been made to review the existing literature in a systematic way. To explain the use of the ‘blind spot’ metaphor in the title, we have added some text to the introduction to explain the use of this metaphor as a way of describing the lack of recognition (including lack of local words to describe the infection), the paucity of existing data, and a neglect of the problem to date. We also return to this point in the discussion, to emphasise the consequences of the blind spot through missed or late diagnosis. The questions posed by the reviewer cannot be completely addressed, due to missing data, but our results do incorporate the lack of missing words to describe the infection in certain languages and the individual and collective responses to infection. These points are more clearly presented as a result of the more robust structure to the results section that we have undertaken in response to feedback from reviewer 1. 

 REVIEWER: First of all, focusing on Africa as a whole (as a target population) is one of the main challenges of the paper. Africa is too diverse to have such generalized ‘lack of knowledge’, ‘misconceptions and prejudices’ regarding HBV. The Moslem North and part of west Africa, the Sub-Saharan Africa, and the relatively wealthy South Africa are different cultures and the knowledge, manifestations, and experience of stigma is bound to be different. The paper can benefit from referring to examples, references in particular places or countries.

 RESPONSE: We agree with this important point, and have added to the discussion accordingly: ‘Sub-Saharan Africa encompasses huge diversity, and as such it would be misleading to assume that we can generalise about culture, beliefs and stigma, or that findings that arise in one setting can be extrapolated to others; the knowledge, manifestations, and experience of stigma is bound to be different between settings. Further work will be needed to develop insights that are relevant to particular populations in order to develop the most effective interventions. Referring to specific examples for particular places or countries is currently difficult in the absence of more published data. However, to understand some of the specific challenges of HBV, we have previously collated individual experiences of HBV from patients, researchers and healthcare workers representing different settings across sub-Saharan Africa (O'Hara et al, doi: 10.1371/journal.pntd.0005842).

 REVIEWER: While the article states the intention to assimilate the available evidence for the nature and impact of stigma on the lives of people with HBV infection and on the community, this does not seem to have been effectively achieved. The paper states very generalized and quite obvious views, which makes it difficult to find new contribution the paper makes to the topic of HBV stigma.

 RESPONSE: We agree with the frustrations expressed here; it is indeed difficult to make advances in the absence of a better literature. However, we see the publication of this article as providing important advocacy for the future development of this field. Although the data may seem ‘generalised and obvious’, they have not currently been translated into any consistent recognition or action. We have added this specifically to the discussion (section headed ‘future aspirations and challenges’) as follows: ‘We see this article as a starting point for this field, as it is currently very difficult to make substantial advances in the absence of better data. By collating the existing literature, we hope to raise the profile of this topic overall, and to highlight specific areas of neglect. This provides a foundation for clinicians and researchers to build on over time.’

 REVIEWER: There is a general tendency to be unspecific regarding manifestation and experience of stigma, by referring to experiences in other conditions – HIV and AIDS, and mental illness, and areas outside Africa. For instance, the authors emphasized lack of knowledge of HBV among different players (health workers, people with HBV infection themselves, community) as potent driver of stigma, and then link the negative effects thereof to prevention, treatment seeking and provision of care.

 RESPONSE: In keeping with comments also raised by reviewer 1, we have improved the clarity around evidence that is HBV-specific, vs. insights that have been gained from sources pertaining to other diseases/infections. The results now contain only HBV-specific findings based on a systematic approach.

 REVIEWER: The authors state that “stereotypes and prejudice may stem from physical differences attributable to the condition, misconceptions associated with fear of contagion, and judgements about routes of transmission, all of which may be underpinned by lack of knowledge … arises from lack of awareness, lack of education and lack of perception … and that healthcare workers provided inaccurate information to those diagnosed by HBV.” While these assertions may be true, the kind of ‘knowledge’ being referred to as lacking in this case, is not clear. First, if there was ‘lack of knowledge’, then what would shape the HBV stigma stated in the paper? The fact that there was stigma, means that there was some form of knowledge; probably ‘inappropriate’ but some knowledge.

 RESPONSE: We have expanded table 2 content regarding ‘lack of knowledge’ to be specific about the gaps in knowledge/understanding, e.g. lack of knowledge ‘about transmission routes’, ‘about treatment availability’, ‘about chronic and asymptomatic nature of HBV infection’, and specific misconceptions e.g. that tobacco causes HBV infection. In this way, we recognise that the lack of knowledge is not absolute but that specific gaps in knowledge can be detrimental.

 REVIEWER: Quite often, there is a tendency to disregard local/indigenous (African) knowledge and understanding of disease and/or health conditions including HBV, and emphasis placed on the biomedical knowledge and quoting sources from the Western World.

 RESPONSE: Thank you for highlighting this omission. As above, we have added specific reference to our previous publication (O'Hara et al, doi: 10.1371/journal.pntd.0005842) in which local experiences are better individually represented. Although most of us currently have affiliations in the UK, it is noteworthy that our authorship list represents personal and individual experience from Uganda, South Africa, Kenya and Zimbabwe. It is difficult to add more robust representation of local beliefs as this is just not apparent in the literature, but we agree that this is a crucial aspiration, and we have therefore added to the discussion to highlight this point, as follows: ‘It is frequently the case that local, indigenous knowledge and understanding of disease and/or health conditions is not well reflected in the published literature’, and subsequently ‘More studies like this are needed in Africa, in order to provide insights into local understanding and beliefs, as they will also help to deploy resources in the most appropriate and effective ways with particular relevance to individual settings’. 

 REVIEWER: Further, generally stigma is known to be characterized by labelling, and social constructs and actions that discount one’s identity (see Goffman: Notes on spoilt Identity). For example, it is not clear what knowledge of HBV bodily signs and symptoms people have, their local names/labels/terminologies, and how this knowledge shapes different forms of stigma. All these seem to have been missing in the literature, which makes it difficult to conclude from the review that people with HBV (in Africa) were actually being stigmatized, leave alone HBV stigma being a blind spot. In other words, the paper does not explicitly show whether or how HBV related stigma (in Africa), is blind spot.

 RESPONSE: We agree that these are important aspirations for ongoing research. We have expanded on the ways in which the ‘blind spot’ metaphor is pertinent (see point above). We have added the reference to Goffman in order to support the point raised about identity, and have expanded the discussion as follows: ‘Findings in Africa demonstrate a general lack of knowledge on HBV, an inability to define the disease, confusion of HBV with other diseases such as malaria and yellow fever, and the association of HBV symptoms with witchcraft or poisoning. Such negative associations have been described to have the potential to ‘spoil the identity’ of the individual: the individual ceases to be perceived as a normal person but rather one who is tainted and discounted from society.  Lack of knowledge or awareness leads to misinformation that feeds stigma and discrimination.’

 REVIEWER: The coping strategies listed in table 3 and discussed in the paper assume existence of particular forms of stigma, that have not been shown to be evident or experienced in Africa. The paper can be improved by linking particular ‘coping’ strategies to particular forms of stigma (in Africa). In fact, in some cases the stigmatized hardly cope, but just endure, and the boundary between coping, and endurance is so blurred.  

 RESPONSE: In the results section, we have improved the layout of this table to reduce duplication (see feedback to reviewer 1). Within the discussion section entitled ‘limitations and caveats’, we have added as follows: ‘Our description of ‘coping strategies’ is an over-simplification of the complex responses that arise as a result of stigma. We recognise that many individuals who suffer stigma may not be able to deploy specific active coping mechanisms, and indeed may simply ‘endure’ their situation’.

 REVIEWER: Discussion:  The claim in the paper, that the data gathered reflect consistent themes of stigma and discrimination … is not adequately illustrated in the results section. Most of the examples given were from Asia and other areas other than Africa, and of other conditions – mental illness and HIV and AIDS, and not HBV, which hardly reflects any consistency at stated.

 RESPONSE: We have removed the use of the description of our results as identifying ‘consistent themes’, and simply state that ‘the data we have gathered reflect stigma and discrimination affecting individuals with chronic viral hepatitis infection’. The new table 1 provides the reader with clearer oversight regarding the geographical origin of the dataset we have used.

 REVIEWER: Limitations: The limitations as stated, considerably weakens the paper. Given the paucity of data, a ‘blind spot on HBV stigma in Africa would be verified by triangulation of information clearly showing what is (in)visible/blindspot, rather than simply interpreting it as lack of literature on the subject matter.

 RESPONSE: We have addressed this point through our specific responses to individual points raised earlier in both reviews. Specifically, this includes a clear comment in the introduction and methods about triangulation of data sources, drawing on HIV, TB and mental illness, and expanded introduction and discussion to include a better description of the ‘blind spot’. It is an interesting point that the blind spot is not as simple as merely a lack of data.

# Peer review: Referee response for version 1

**DOI:** 10.21956/wellcomeopenres.15534.r32087

**Published:** 2018-05-10

**Authors:** Laura Nyblade, Melissa Stockton

**Affiliations:** 1 RTI International, Washington, DC, USA; 2 University of North Carolina, Chapel Hill, NC, USA

Summary:  The article presents findings from a systematic review on HBV stigma. The authors recognize the large burden of HBV globally and the potential for stigma to hamper prevention, diagnosis and treatment. They unpack the lack of knowledge underpinning the stigmatization of HBV as well as some factors associated with stigmatization of HBV. They also present findings on how HBV stigma impacts health care, familial and social relationships, employment, mental health, and behavior. The authors also describe strategies individuals use to cope with or manage HBV stigma and interventions to address HPV stigma. Finally, the authors conclude by identifying gaps in the literature, particularly a paucity of research conducted in Africa, and provide guidance for future research.

**Specific Comments to explain ratings:**

With respect to rating for Question 1 (partly): The rationale for the paper is clear, however the objectives of this paper are less clear. We are uncertain what the word assimilate means with respect to the literature.  Did the authors mean to use this term?  If so, it may be helpful to explain it further.    As well, it’s unclear if the authors are going to abstract suggestions to address stigma and discrimination from the included articles or if they are going to use their findings to provide suggestions, drawing on literature from other stigmatized conditions. It is also unclear from the results section, and in particular the literature included in the tables, if this literature review is restricted to HBV stigma, or also includes HIV and mental health stigma (in which case the review is not extensive enough).   The supplementary material showing the search strategy indicates it is restricted to HBV stigma, yet the results section suggests otherwise.   The paper would be strengthened by a clearer statement of the objectives and then ensuring that those objectives are adhered to throughout.  

With respect to rating for Question 2 (partly): The authors do not include their specific inclusion/exclusion criteria, which relates to the point above with respect to the mixing of what looks like included literature from the search, with other literature from HIV and Mental Health in the results section. It is also unclear how “factors associated with stigma,” “impact of stigma on the lives of people with HBV,” and “proposed interventions to tackle stigma in the society” were systematically captured. There is one article included in the data abstraction supplementary file (Cochrane et al 2016 ^1^) for which “not/applicable” is listed for all of the data abstraction categories related to stigma. It is unclear why this article is included.

 More detail is needed on the process of reviewing and selecting which abstracts went to full review and then which articles were included.   Was this done by one person?   Two people who then compared results with a third as tie breaker in cases of disagreement?   This information will help the reader assess the strength of the review.

 A standard figure showing the two searches graphically would be helpful for the reader (how many initial abstracts, how many of those went to full review and then how many of those were included).

With respect to rating for Question 4 (partly):  The manuscript would benefit from the authors including a discussion on risk of bias in their process and review. They do not mention bias or describe any methods used for assessing risk of bias of individual included studies.   Other limitations that need to be mentioned include that many of the findings presented in the results section come from a single article (e.g. in most of the tables, each point often only has one reference attached to it, and in some cases not even a reference from the search itself).   

**Other comments:**

Abstract:  The authors state that only one study was conducted in Africa, while in the limitations it states that two studies were conducted in Africa. 

Overall language comment:  Please do not use HIV/AIDS, it is considered stigmatizing.  UNAIDS language guidelines recommend using HIV, HIV and AIDS, or HIV or AIDS, depending on the context.   Using HIV/AIDS implies HIV=AIDS and that one cannot live a long, healthy and productive life with HIV.   

Methods:   See comment above on explanation for rating for Question 2.  

Results:   The results section would be strengthened by presenting only the results of the search in the results section and keeping discussion (and inclusion of non-HBV stigma literature) to the discussion section.   The results section often seems more like a discussion section and it is sometimes difficult to tell what findings are coming from the HBV stigma literature review, versus what are coming from other articles not included in the review (e.g. some of the findings presented in tables are from HIV, mental health or general stigma literature), or are views/conclusion the authors have drawn. For example, in the 4 ^th^ paragraph of the “Factors associated with creating or perpetuating stigma for individuals with HBV Infection” section, the authors hypothesize about why a certain trend is observed. As well, the “coping strategies and interventions for HBV stigma” section is almost entirely a discussion of coping strategies that have been used to tackle stigma in other fields or interventions that have been used to address stigma related to diseases other than HBV. While pertinent, this information probably belongs in the discussion section.

 The results section would benefit from a clearer organizational structure and ensuring only results from the HBV literature are included. For example,

- “Factors associated with creating or perpetuating stigma for individuals with HBV Infection”            
  - It seems the first section” is trying to unpack factors driving or causing stigma and factors associated with higher levels of stigma. However, this section also includes some behavioral impacts, such as pursing traditional or religious intervention at the individual level, which could be considered “impact on access to care”
  - At the health care level, providing inaccurate information and the provision of inappropriate reassurance or over emphasis of potential complications seem to be the result of lack of knowledge, rather than evidence of lack of knowledge, that could be considered “impact on access to care” or “impact on quality of care.”
  - The text notes stigma among policy makers can be problematic, but this isn’t supported by evidence in the table.
  - Table 1:   Unclear if these factors are actually coming from the HBV literature or not, given the inclusion of HIV articles in the table that from the supplementary material describing the literature search, were not included.   For example references 40, 41.
  - Table 2:
    1. The “Impact on mental health” section of the table seems to have some redundant items, particularly around guilt and shame
    2. The “Impact on social behavior” section of the table seems to largely encompass manifestations of stigma, but not necessarily social behaviors. It is unclear what is meant by “suffer feelings of “passivity” because of lack of treatment, or cost of treatment, which results in missing…”
    3. The “relationship with education/knowledge” section seems to almost entirely overlap with the previous table, highlighting factors associated with stigma. However, it is interesting to highlight that stigma may drive lack of knowledge just as lack of knowledge may drive stigma. However, this might be better said in the text.
    4. All references to articles that were not part of the actual HBV stigma review should be removed.   This includes references 40, 41, 57,58, 59.   The articles should instead be referred to in the discussion section if the authors feel they are important to discuss around a key result.
- Coping strategies and interventions for HBV stigma”
   
  - Table 3
    1. The title “Coping strategies associated with negative influence” is unclear. It seems like this bullet is for interventions.
    2. All references that are not coming directly from the review need to be removed.  Specifically, #’s 40,48, 57, 58, 70
- The table included in the supplementary material showing the articles should be included within the body of the article in a reduced format, given it is central to the review process. 
    

Discussion:   The discussion section paragraph reads more like a conclusion paragraph, while there is much discussion embedded in the results section.   Moving the discussion from the results into the discussion section will strengthen both the results and discussions section and also make clearer to the reader what is emanating directly from the review of the HBV literature versus what the authors are supplementing from other fields, because of the sparse HBV stigma literature.    The discussion section could be expanded by discussing what the gaps are that were found—and then using the literature from other fields to suggest how those gaps might be filled etc. 

Limitations: In addition to comments above to Q.4, it would be appropriate to discuss the strength of findings/quality of included articles. Was any rating of the quality of articles conducted? We also find the statement at the end of the limitations section that literature from HIV, TB and mental illness was drawn on, because of the limitations of the literature found to be confusing. The fact that this was done, muddies the methods and results sections. If the literature review is only focused on HBV stigma, no matter how scarce the literature is, only this literature should be drawn on/presented in the results section. Other literature can/should be discussed in the discussion section. This is conflating of the results coming from the search itself, with ‘supplementary’ literature from other fields in the result section is problematic. Please move all discussion of other literature not coming from the literature search on HBV to the discussion section.  

Conclusions:  The conclusions state stigma may disrupt adherence, however, adherence to treatment is not discussed in the results. However, hampered adherence is listed as an impact of stigma in the abstraction table for the Huang *et al *2016 ^2^ article.

We have read this submission. We believe that we have an appropriate level of expertise to confirm that it is of an acceptable scientific standard, however we have significant reservations, as outlined above.

## References

[1] CochraneACollinsPHorwoodJP: Barriers and opportunities for hepatitis B testing and contact tracing in a UK Somali population: a qualitative study. *Eur J Public Health*.26(3) : 10.1093/eurpub/ckv236 389-95 10.1093/eurpub/ckv236 26896472

[2] HuangJGuanMLBalchJWuERaoHLinAWeiLLokAS: Survey of hepatitis B knowledge and stigma among chronically infected patients and uninfected persons in Beijing, China. *Liver Int*.36(11) : 10.1111/liv.13168 1595-1603 10.1111/liv.13168 27206379

# Response

**Published:** 2018-08-14

**Authors:** Philippa Matthews

**Affiliations:** Nuffield Department of Medicine, Peter Medawar Building for Pathogen Research, University of Oxford, UK

REVIEWER: The rationale for the paper is clear, however the objectives of this paper are less clear. We are uncertain what the word assimilate means with respect to the literature.  Did the authors mean to use this term?  If so, it may be helpful to explain it further.   

AUTHOR RESPONSE: As defined by the Oxford English dictionary, the term ‘assimilate’ means to ‘take in and understand fully (information or ideas)’. We have added to the final paragraph of the introduction to clarify this: ‘Our approach was to gather relevant information from the published literature, to curate it in order to be able to unify key messages, and to highlight gaps where future work is still needed’. By strengthening our consistent adherence to the principles of a systematic literature review throughout the paper (addressed in more detail in the points below), our objectives are now set out in a more transparent way.

REVIEWER: As well, it’s unclear if the authors are going to abstract suggestions to address stigma and discrimination from the included articles or if they are going to use their findings to provide suggestions, drawing on literature from other stigmatized conditions.

RESPONSE: We have used the reviewer’s suggested text at the end of the introduction, to describe both of these approaches, adding: ‘We have been able to develop suggestions to address HBV stigma and discrimination from the existing literature, but in light of the paucity of HBV-specific studies, we have also drawn on literature from other stigmatized conditions’.

REVIEWER: It is also unclear from the results section, and in particular the literature included in the tables, if this literature review is restricted to HBV stigma, or also includes HIV and mental health stigma (in which case the review is not extensive enough).   The supplementary material showing the search strategy indicates it is restricted to HBV stigma, yet the results section suggests otherwise.   The paper would be strengthened by a clearer statement of the objectives and then ensuring that those objectives are adhered to throughout.  

RESPONSE: The primary focus of our review is HBV stigma, however in the absence of clear literature on this topic we have also drawn on wider literature about stigma in other conditions. We agree that this was not sufficiently clear or consistent as an approach in our initial version, and have amended the article accordingly as follows:

1. Introduction: we have amended the final paragraph to set out the approach more clearly, describing a systematic approach, curation to unify key themes, and highlighting gaps where future work is still needed. We also explain the need to triangulate our approach by drawing on resources from other stigmatized conditions including HIV, TB and mental illness to inform the discussion.
2. Methods: we have made a clear distinction between the systematic literature review undertaken for HBV stigma, versus a more general use of literature on other conditions to provide additional breadth and context.
3. Results: these are now clearly presented as pertaining only to the systematic literature review, reflecting HBV-specific data (see opening sentence of results section).
4. Discussion: we have used this section to reference the wider literature on stigma, using HIV, mental health and TB as examples.

 

REVIEWER: The authors do not include their specific inclusion/exclusion criteria, which relates to the point above with respect to the mixing of what looks like included literature from the search, with other literature from HIV and Mental Health in the results section.

RESPONSE: We have tackled this in a number of ways:

1. We have improved a description of our search strategy to make clear the specific focus on HBV stigma. This expanded section now reads as follows: ‘we undertook a systematic literature search in PubMed; our search strategy focused entirely on the evidence surrounding stigma in HBV’. 
2. We have moved the PRISMA flow diagram into the paper as Fig 1, thus providing the reader with a clear summary of our approach.
3. We have divided our methods surrounding the literature search into two sections, first ‘ **Search strategy: systematic literature review’**, and second ‘ **Search strategy: other relevant resources’**. This makes a distinction between the HBV component of the research, that has been conducted as a robust systematic literature review, versus other supporting references that ‘were not identified through a formal systematic review, but were identified as relevant sources from robust, peer reviewed literature.’

 

REVIEWER: It is also unclear how “factors associated with stigma,” “impact of stigma on the lives of people with HBV,” and “proposed interventions to tackle stigma in the society” were systematically captured. There is one article included in the data abstraction supplementary file (Cochrane et al 2016) for which “not/applicable” is listed for all of the data abstraction categories related to stigma. It is unclear why this article is included.

RESPONSE: We have included a description of representing our results as a number of themes. This now reads ‘We used our research question to group data into four major themes: factors underpinning stigma in HBV infection; evidence for the impact of stigma; coping strategies for individuals with HBV infection’. We have revised the metadata for all included studies: Cochrane et al 2016 does meet our inclusion criteria and we have included information on stigma extracted from this study in the results.

REVIEWER: More detail is needed on the process of reviewing and selecting which abstracts went to full review and then which articles were included.   Was this done by one person?   Two people who then compared results with a third as tie breaker in cases of disagreement?   This information will help the reader assess the strength of the review.

RESPONSE: The initial review of abstracts was performed by one individual according to prospectively agreed, objective criteria, as set out in the PRISMA document. Two individuals contributed to the next stage of the review; this is now included in methods.

   

REVIEWER: A standard figure showing the two searches graphically would be helpful for the reader (how many initial abstracts, how many of those went to full review and then how many of those were included).

RESPONSE: This was originally in supplementary data, but we have now included a PRISMA flow diagram as fig 1 in the manuscript, as well as adding the relevant numbers to the text.

REVIEWER: The manuscript would benefit from the authors including a discussion on risk of bias in their process and review. They do not mention bias or describe any methods used for assessing risk of bias of individual included studies.  

RESPONSE: We have now added a ‘risk of bias assessment’ approach; for qualitative studies we used the qualitative appraisal checklist by NICE public health guidance and for quantitative studies we used the Centre of Evidence based Management checklist. This has been added to the methods, and has informed a new paragraph in the results entitled ‘quality of. We have added new suppl tables 3 and 4 containing the detailed results of this assessment for each paper. Under the section of limitations/ caveats, we also describe some of the shortfalls of the included studies, recognising the paucity of the literature overall and in particular for Africa.

REVIEWER: Other limitations that need to be mentioned include that many of the findings presented in the results section come from a single article (e.g. in most of the tables, each point often only has one reference attached to it, and in some cases not even a reference from the search itself).

RESPONSE: We have rectified this. The all results presented in the tables include only studies on HBV stigma identified from our systematic literature search. Where possible, we have added more than one reference for each point that is included; this now represents a comprehensive summary of all 34 included studies. However, we recognise that the literature is limited and therefore some points can only be supported by only one citation. 

   

REVIEWER: Abstract:  The authors state that only one study was conducted in Africa, while in the limitations it states that two studies were conducted in Africa. 

RESPONSE: We have corrected accordingly (two studies were conducted in Africa). We have also improved the clarity of this in the new table 1, by dividing the literature by geographical region so it is easy to pick out the studies that are set in any given location.

REVIEWER: Please do not use HIV/AIDS, it is considered stigmatizing.  UNAIDS language guidelines recommend using HIV, HIV and AIDS, or HIV or AIDS, depending on the context.   Using HIV/AIDS implies HIV=AIDS and that one cannot live a long, healthy and productive life with HIV.   

RESPONSE: Thank you for this feedback, we have corrected accordingly (although please note that several of our references use this abbreviation in their titles).

REVIEWER: The results section would be strengthened by presenting only the results of the search in the results section and keeping discussion (and inclusion of non-HBV stigma literature) to the discussion section. The results section often seems more like a discussion section and it is sometimes difficult to tell what findings are coming from the HBV stigma literature review, versus what are coming from other articles not included in the review (e.g. some of the findings presented in tables are from HIV, mental health or general stigma literature), or are views/conclusion the authors have drawn. For example, in the 4 ^th^ paragraph of the “Factors associated with creating or perpetuating stigma for individuals with HBV Infection” section, the authors hypothesize about why a certain trend is observed.

RESPONSE: We have taken a careful approach to improving this; our results tables now cite only references that are derived from the systematic literature review. We have now moved all hypotheses and references to other diseases into the discussion section.

REVIEWER: As well, the “coping strategies and interventions for HBV stigma” section is almost entirely a discussion of coping strategies that have been used to tackle stigma in other fields or interventions that have been used to address stigma related to diseases other than HBV. While pertinent, this information probably belongs in the discussion section.

RESPONSE: We agree; we have trimmed the inclusion of coping strategies out of the results section and now feature more of this text in the discussion.

REVIEWER: The results section would benefit from a clearer organizational structure and ensuring only results from the HBV literature are included. For example,

- “Factors associated with creating or perpetuating stigma for individuals with HBV Infection”            
  - It seems the first section” is trying to unpack factors driving or causing stigma and factors associated with higher levels of stigma. However, this section also includes some behavioral impacts, such as pursing traditional or religious intervention at the individual level, which could be considered “impact on access to care”
  - At the health care level, providing inaccurate information and the provision of inappropriate reassurance or over emphasis of potential complications seem to be the result of lack of knowledge, rather than evidence of lack of knowledge, that could be considered “impact on access to care” or “impact on quality of care.”
  - The text notes stigma among policy makers can be problematic, but this isn’t supported by evidence in the table.

 

RESPONSE: As per our responses to points above, we have improved the results section to make it more consistent and relating only to the HBV literature in the papers we identified through a systematic approach. Most of the additional text has been relocated to the discussion section. We have revisited the classification of each individual point into four different themes (represented by tables 2-5), although there is naturally some cross-referencing between these closely related areas. We have removed the inclusion of stigma among policy makers as we agree it is not sufficiently supported by the literature that we have identified.

REVIEWER: Table 1:  Unclear if these factors are actually coming from the HBV literature or not, given the inclusion of HIV articles in the table that from the supplementary material describing the literature search, were not included.   For example references 40, 41. 

RESPONSE: We have corrected the tables, all the information included are from studies on HBV stigma identified from systematic literature review, and provided table 1 to summarise the literature on which we have based all other results tables.

REVIEWER: Table 2: The “Impact on mental health” section of the table seems to have some redundant items, particularly around guilt and shame.

RESPONSE: We agree, and have collated the relevant points and references into a single statement supported by several references.

REVIEWER: The “Impact on social behavior” section of the table seems to largely encompass manifestations of stigma, but not necessarily social behaviors. It is unclear what is meant by “suffer feelings of “passivity” because of lack of treatment, or cost of treatment, which results in missing…”

RESPONSE: We have changed this sub-heading from ‘impact on social behaviour’ to ‘impact on social interactions’ which better encompasses the points that follow. We have also rephrased the statement to describe instead a ‘passive’ attitude towards treatment.

REVIEWER: Table 2: The “relationship with education/knowledge” section seems to almost entirely overlap with the previous table, highlighting factors associated with stigma. However, it is interesting to highlight that stigma may drive lack of knowledge just as lack of knowledge may drive stigma. However, this might be better said in the text.

RESPONSE: We agree that the sections referring to knowledge and education in Tables 1 and 2 can be assimilated, and have moved all of this into a single section in Table 1. We agree with this interesting reflection raised by the reviewer, which we have added to the discussion as follows: ‘Education clearly provides a very important role in reducing discrimination and stigma; it is interesting to note that stigma may drive lack of knowledge, just as lack of knowledge may drive stigma.’

REVIEWER: All references to articles that were not part of the actual HBV stigma review should be removed.   This includes references 40, 41, 57,58, 59.   The articles should instead be referred to in the discussion section if the authors feel they are important to discuss around a key result.

RESPONSE: As above, we have removed any references that are not HBV-specific from the tables.

REVIEWER: Coping strategies and interventions for HBV stigma - Table 3:1. The title “Coping strategies associated with negative influence” is unclear. It seems like this bullet is for interventions.

RESPONSE: We agree this was not clearly presented; we have changed this table to remove the first rows and to remove the idea of ‘coping strategies associated with negative influence’. The point we made here is now included in the previous table, listed simply as ‘emotional responses including grief, denial, anger and aggression’.

REVIEWER: All references that are not coming directly from the review need to be removed.  Specifically, #’s 40,48, 57, 58, 70

RESPONSE: This has been done, as per responses to points above.

REVIEWER: The table included in the supplementary material showing the articles should be included within the body of the article in a reduced format, given it is central to the review process.

RESPONSE: Thank you – this is a good suggestion. We have added a reduced version of this table as Table 1 in the results section, grouping the results by geographical area. We have also retained the link to a Suppl data file in order to provide interested readers with further details.

REVIEWER: Discussion:   The discussion section paragraph reads more like a conclusion paragraph, while there is much discussion embedded in the results section.   Moving the discussion from the results into the discussion section will strengthen both the results and discussions section and also make clearer to the reader what is emanating directly from the review of the HBV literature versus what the authors are supplementing from other fields, because of the sparse HBV stigma literature.    The discussion section could be expanded by discussing what the gaps are that were found—and then using the literature from other fields to suggest how those gaps might be filled etc. 

RESPONSE: We have taken a careful approach to dividing the results and discussion. The results are now condensed largely into a series of tables, presenting only objective findings from the systematic literature review. Other points, building on literature for other diseases, have now all been moved into the discussion.

REVIEWER: Limitations: In addition to comments above, it would be appropriate to discuss the strength of findings/quality of included articles. Was any rating of the quality of articles conducted? We also find the statement at the end of the limitations section that literature from HIV, TB and mental illness was drawn on, because of the limitations of the literature found to be confusing. The fact that this was done, muddies the methods and results sections. If the literature review is only focused on HBV stigma, no matter how scarce the literature is, only this literature should be drawn on/presented in the results section. Other literature can/should be discussed in the discussion section. This is conflating of the results coming from the search itself, with ‘supplementary’ literature from other fields in the result section is problematic. Please move all discussion of other literature not coming from the literature search on HBV to the discussion section.

RESPONSE: As above, this has been done.

  

REVIEWER: Conclusions:  The conclusions state stigma may disrupt adherence, however, adherence to treatment is not discussed in the results. However, hampered adherence is listed as an impact of stigma in the abstraction table for the Huang *et al *2016 article.

RESPONSE: Specific reference to treatment adherence is now included in the results section (table 3), supported by relevant references. These do not now include the paper by Huang et al, as this study did not provide primary data on adherence (it is mentioned briefly as a discussion point only, supported by other references).
